# Allosterism in the PDZ Family

**DOI:** 10.3390/ijms23031454

**Published:** 2022-01-27

**Authors:** Amy O. Stevens, Yi He

**Affiliations:** Department of Chemistry and Chemical Biology, The University of New Mexico, Albuquerque, NM 87131, USA; ao630@unm.com

**Keywords:** PDZ domain, allosterism, dynamic allostery, key residues

## Abstract

Dynamic allosterism allows the propagation of signal throughout a protein. The PDZ (PSD-95/Dlg1/ZO-1) family has been named as a classic example of dynamic allostery in small modular domains. While the PDZ family consists of more than 200 domains, previous efforts have primarily focused on a few well-studied PDZ domains, including PTP-BL PDZ2, PSD-95 PDZ3, and Par6 PDZ. Taken together, experimental and computational studies have identified regions of these domains that are dynamically coupled to ligand binding. These regions include the αA helix, the αB lower-loop, and the αC helix. In this review, we summarize the specific residues on the αA helix, the αB lower-loop, and the αC helix of PTP-BL PDZ2, PSD-95 PDZ3, and Par6 PDZ that have been identified as participants in dynamic allostery by either experimental or computational approaches. This review can serve as an index for researchers to look back on the previously identified allostery in the PDZ family. Interestingly, our summary of previous work reveals clear consistencies between the domains. While the PDZ family has a low sequence identity, we show that some of the most consistently identified allosteric residues within PTP-BL PDZ2 and PSD-95 PDZ3 domains are evolutionarily conserved. These residues include A46/A347, V61/V362, and L66/L367 on PTP-BL PDZ2 and PSD-95 PDZ3, respectively. Finally, we expose a need for future work to explore dynamic allostery within (1) PDZ domains with multiple binding partners and (2) multidomain constructs containing a PDZ domain.

## 1. Background

Allosterism is a phenomenon where communication exists within a biological macromolecule between the ligand-binding site and a distal region. While allosteric effects were initially attributed only to structural changes, Cooper and Dryden [1] made a case for a new model of allosterism that addresses conformational dynamics. In this new model, communication is not limited to discrete structural changes but instead can point to a global dynamical shift. While the traditional view of allostery points to a clear mechanism of cellular regulation via structural changes, the proposition of regulation via dynamic allostery is entropically driven as increased global fluctuations encourage thermodynamically favorable reactions. Various reviews have excellently summarized our understanding of allosterism [2,3,4,5,6]. While many proteins use dynamic allosterism to regulate cellular processes, the PDZ family is a classic example of dynamic allostery in small modular domains.

**Figure 1:**
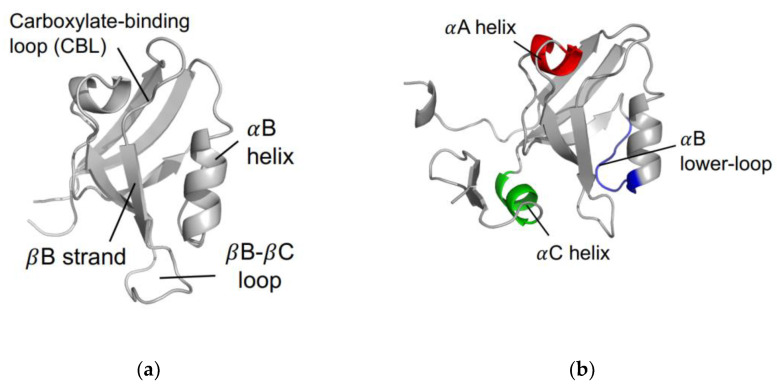
The PDZ domain. (**a**) Canonical fold of the PDZ family. Shown here is PTP-BL PDZ2 (PDB ID: 1GM1 [19]). (**b**) Proposed regions of dynamic allostery on the PDZ domain, including the αA helix (red), the αB lower-loop (blue), and the αC helix (green). Note that the αC helix is unique to PSD-95 PDZ3, as shown here (PDB ID: 1BE9 [17]).

The PDZ family comprises 268 domains in 151 unique human proteins [7]. The sheer number of PDZ domains opens a wide variety of cellular processes that include the PDZ family. Most often, PDZ domains are involved in regulating signaling pathways [8,9,10,11,12,13]. For example, PDZ domains have key roles in managing cell polarity; regulating tissue growth and development; trafficking of membrane protein receptors and ion channels; and regulating cellular pathways [14,15,16]. In addition to a broad variety of biological functions, the PDZ family has a relatively high level of variation within the primary sequence of each domain. Despite a relatively low sequence identity, the secondary structure within the PDZ family is highly conserved. A canonical PDZ domain comprises six β-strands and two α-helices. It has a single binding site in the hydrophobic groove between the αB-helix and the βB-strand [17], as shown in Figure 1a. Most commonly, PDZ domains interact with the final three to five C-terminal residues of target proteins via the carboxylate binding loop that is defined by the conserved χ-φ-Gly-φ motif, where χ is any residue and φ is any hydrophobic residue [18].

As key players in regulating signaling pathways, the PDZ family propagates signals via dynamic allosterism. Various groups have revealed how highly conserved protein-protein interactions in the PDZ binding pocket propagate allosteric effects through the PDZ domain [19,20,21,22,23,24,25,26,27,28,29,30,31,32,33]. Understanding the origin, the destination, and the pathway of the signal could greatly aid our understanding of how the cell uses allosterism in cellular regulation. Ultimately, this understanding of a domain with such a broad biological role could open the door to new targets in drug development. Here, we will review previous work that has identified dynamic allostery in the PDZ family while specifically noting key residues of the PDZ domain that have consistently been identified as universal regions of dynamic allostery. 

In 1999, Lockless and Ranganathan brought allostery in the PDZ family to attention. Suspecting that allosteric networks are evolutionarily conserved, they proposed that such networks could be statistically predicted using multiple sequence alignment (MSA) [20]. Lockless et al. performed MSA on 274 eukaryotic PDZ domains to introduce two networks of energetically coupled residues that may be responsible for the propagation of allostery throughout the PDZ domain. This original study opened a new door to explore allostery within the PDZ family. As various groups followed their footsteps, the results of dynamic allostery have been wildly different. Among experimental approaches, NMR experiments have been used as a primary tool to identify allostery as NMR data can describe both protein structure and protein dynamics. For example, ^15^N/^13^C [19,27,28,34,35,36,37] and ^1,2^H-methyl [27,35,36,37] spin relaxation data have been used to reveal backbone and side-chain dynamics, respectively. Relaxation data produce two useful parameters (S^2^ and τ_e_) that can describe protein dynamics in the picosecond to nanosecond timescale. The S^2^ parameter describes the amplitude of a bond’s fluctuations, where a value of 1 corresponds to complete rigidity and a value of 0 corresponds to complete flexibility. The τ_e_ parameter describes the timescale of these motions. Several groups have wonderfully summarized allostery in the PDZ family [38,39,40,41,42,43,44]. Here, we will specifically review three proposed regions of the PDZ with dynamic allostery, including the αA helix, αB lower-loop, and the αC helix (PSD-95 PDZ3). We have chosen to focus on these three regions because the allosteric residues identified by previous work are most commonly found here. Thus, the greatest agreement across multiple works occurs at these regions. The three regions of interest are shown in Figure 1b. The purpose of this review is to summarize the observed allosteric effects in three well-studied PDZ domains (PTP-BL PDZ2, PSD-95 PDZ3, and Par6 PDZ) by providing specific key residues consistently noted in each study. This summary can be used as an index for future researchers looking to trace previously identified dynamic allosterism in the PDZ family.

### 1.1. The αA Helix

Lockless and Ranganathan’s original study identified the αA helix in the network of residues propagating dynamic allostery through PDZ. Since this initial hypothesis, more than a dozen publications have pointed to the αA helix as a key player in dynamic allostery. These studies have recognized αA allostery by utilizing experimental and computational approaches, including nuclear magnetic resonance (NMR) [19,25,26,27,28,30,45], all-atom molecular dynamics (MD) [21,29,31,46,47], Monte-Carlo sampling [48], anisotropic thermal diffusion (ATD) [33,49], normal mode analysis (NMA) [22], and perturbation response scanning (PRS) [50]. Collectively, the various approaches have primarily focused on three PDZ domains: PTP-BL PDZ2, PSD-95 PDZ3, and Par6 PDZ.

#### 1.1.1. Agreement between Experimental and Computational Techniques: PTP-BL PDZ2

NMR experiments have identified αA allostery triggered by ligand binding in PTP-BL PDZ2. In 2002, Walma et al. noted chemical shifts at the αA helix of PTP-BL PDZ2 upon binding to human Fas receptor and RIL C-terminal peptides [19]. Two years later, Fuentes et al. observed dynamics changes at the αA helix while bound to RA-GEF2 peptide by specifically noting significant chemical shifts at residues A39 and V40 [27]. They note a clear pathway from the binding pocket to the αA helix as the helix forms a van der Waals surface with I20, which is directly involved in ligand binding. In a continuing study, Fuentes et al. performed point mutations to previously identified key binding residues such as I20 on the βB strand [35]. NMR experiments showed that the I20F mutation led to significant dynamics changes at I41 and A46 of the αA helix. In summary, these studies applied NMR experiments to identify αA allostery in PTP-BL PDZ2. Furthermore, Fuentes et al. [27,35] proposed signal transduction pathways from the binding pocket to the αA helix.

Computational techniques have been able to reproduce the experimentally observed αA allostery in PTP-BL PDZ2 via MD [29,30,31], Monte-Carlo sampling [48], PRS [50], and ATD [33]. Here, we will first explore αA allostery in PTP-BL PDZ2 from the viewpoint of equilibrium MD. Dhulesia et al. [29] performed 16 replicas of 2 ns MD simulations restrained with NMR data [27,51,52] using the CHARMM22 force field. Upon RA-GEF2 binding, the motion of the αA helix (A39, l40, A45, and A46) decreases and couples with the motion of the βB stand. They propose a pathway of transduction from l35 to V37 to I52, where I35 directly forms interactions with RA-GEF2. While Kong et al. did not apply experimental restraints, their 10 replicas of 5 ns MD simulations with the CHARMM27 force field identified a signal transduction pathway at the binding pocket and extending along the αA helix [46]. In the complex state, residues S17 and L18 of the carboxylate binding loop are coupled with residues I41, Q43, G44, A45, A46, E47, and S48 of the αA helix. Notably, they propose that the distal proximity of the carboxylate binding loop, and the αA helix may serve as a direct pathway for the transduction of the allosteric signal. Similarly to Kong et al., Morra et al. were also able to reproduce allostery at the αA helix without the use of experimental restraints [31]. Morra et al. used the GROMACS force field to perform two 400 ns trajectories. Upon binding to RA-GEF2, there are significant changes in the dynamic fluctuations of the αA helix (K38, A39, V40, P42, Q43, G44, and G50). While all three equilibrium MD simulations point to allostery at the αA helix, the allostery itself differs. Where Dhulesia notes a decrease in fluctuation at the αA helix, Morra et al. reports an increase. Furthermore, Kong et al. defines allostery at αA as a coupling of motion to the binding pocket rather than an increase or decrease in motion upon ligand binding.

**Figure 2:**
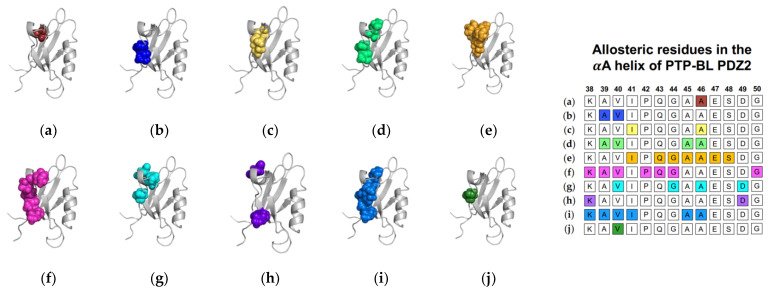
Ligand-induced dynamic allostery at the αA helix in the PTP-BL PDZ2 (PDB ID: 3PDZ [51]) domain as observed in experimental and computational studies. (**a**) NMR chemical shifts by Walma et al. [19]; (**b**) NMR chemical shifts by Fuentes et al. [27]; (**c**) point mutations and NMR chemical shifts by Fuentes et al. [35]; (**d**) equilibrium MD simulations constrained with NMR data by Dhuelsia et al. [29]; (**e**) equilibrium MD simulations by Kong et al. [46]; (**f**) equilibrium MD simulations by Morra et al. [31]; (**g**) Monte-Carlo sampling Cilia et al. [48]; (**h**) protein structure network and elastic network model (PSN-ENM) by Raimondi et al. [53]; (**i**) perturbation response scanning (PRS) by Gerek et al. [50]; and (**j**) rigid-residue scanning (RRS) by Kalescky et al. [54]. The neighboring table displays the sequence fragment of the αA helix in the PTP-BL PDZ2 domain with allosteric residues colored accordingly. Note that the colored residues in the table directly correspond to the colored residues in the structural representations on the left.

In addition to equilibrium MD, Cilia et al. used Monte-Carlo sampling (751,500 conformations) to identify a change in dynamics at the αA helix (V40, G44, A46, and D49), without commenting on whether fluctuations increased or decreased [48]. Furthermore, non-equilibrium simulation techniques including ATD, PSN-ENM, and RRS have been used to trace global energy flow from a selected residue. Gerek et al. applied perturbation response scanning (PRS) to identify dynamically important residues [50]. “Hot residues” (K38, A39, V40, I41, A45, and A46 of the αA helix) had the highest mean square fluctuation response to the perturbation. In 2013, Raimondi et al. applied a combined strategy of Protein Structure Network (PSN) and Elastic Network Model (ENM) to identify communication pathways within PTP-BL PDZ2 [53]. Their pathway included K38 and D49. Lastly, Kalescky et al. combined rigid-residue molecular dynamics with entropy analysis to explore the role of each residue in the global dynamics of PTP-BL PDZ2 [54]. They identified several residues including V40 of the αA helix that have a key role in the allosteric network. In summary, experimental and computational techniques have both identified αA allostery in PTP-BL PDZ2. Each study described above is summarized in Figure 2. Each study is represented by a corresponding PTP-BL PDZ2 domain with the identified allosteric residues colored and shown in a sphere model. The neighboring table titled, “Sequence fragment of the αA helix in PTP-BL PDZ2”, is color coordinated with each structure to visualize any residue-level agreement between previous studies. Our summary points to the importance of V40 and A46 in ligand-induced dynamic allosterism at the αA helix.

#### 1.1.2. Computational Conclusions Lacking Experimental Support: PSD-95 PDZ3

While computational techniques have pointed to ligand-induced αA allostery in PSD-95 PDZ3, these results have not been consistently confirmed experimentally. As the first PDZ domain to have its structure characterized by x-ray crystallography [10,17], PSD-95 PDZ3 is arguably the most well-studied domain in the PDZ family. Original NMR experiments exploring the structure of PSD-95 PDZ3 comment on the unchanging backbone structure from the unbound state to the bound state [17]. Sequential NMR studies have focused on allosterism at the αC helix rather than the αA helix [36,37,55]. While exploring the αC helix, Zhang et al. show that phosphorylation at αC induced significant chemical shifts at αA [37], thus supporting αA allostery induced by perturbations at αC but failing to confirm αA allostery due to ligand binding. Meanwhile, computational efforts have consistently pointed to ligand binding inducing dynamic allostery at the αA helix.

Ligand-induced αA allostery in the PSD-95 PDZ3 domain has been identified using equilibrium MD [21,31], nonequilibrium MD [47], and other computational techniques [49,50,56,57,58]. In 2005, Ota et al. applied ATD to measure anisotropic thermal diffusion of kinetic energy throughout the PSD-95 PDZ3 domain [49]. After applying thermal energy to H372 on the αB helix, Ota et al. observed a distinct propagation of energy to I341 and A347 of the αA helix. In 2010, Ho et al. applied rotamerically induced perturbations to identify residues in the αA helix (P346, A347) that were correlated to key residues in ligand binding [59]. Again in 2010, Du et al. applied a computational approach called amino acid position conservative-mutation correlation analysis (CMCA) to identify evolutionary networks [58]. They found that mutating residues on the αA helix (L342, A343, G344, A347, D348, and G351) regulates the binding affinity between PSD-95 PDZ3 and its ligand. Their results point to an allosteric mechanism by which interactions at the αA helix regulate ligand binding. In another unique computational technique, Gerek et al. applied perturbation response scanning (PRS) to identify dynamically important residues, such as G345 P346, A347, L353, and R354 of the αA helix [50]. In 2012, McLaughlin et al. performed a complete single-mutation scan coupled with statistical coupling analysis (SCA) [56]. While various mutations to binding pocket residues affected ligand binding, mutations to the αA helix (A347 and L353) also significantly affected binding.

**Figure 3:**
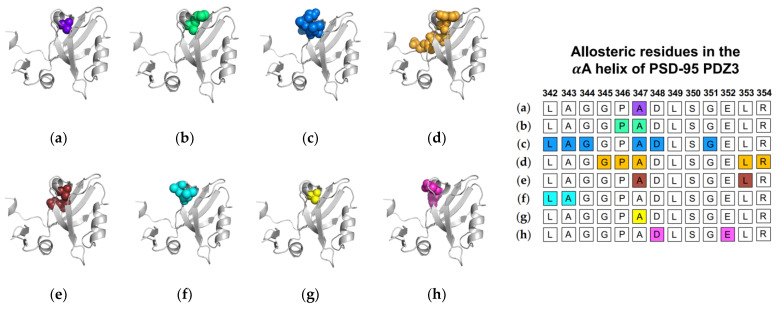
Ligand-induced dynamic allostery at the αA helix in the PSD-95 PDZ3 domain (PDB ID: 1BE9 [17]) as observed in computational studies. (**a**) Anisotropic thermal diffusion (ATD) by Ota et al. [49]; (**b**) rotamerically induced perturbations (RIP) by Ho et al. [59]; (**c**) conservative-mutation correlation analysis (CMCA) by Du et al. [58]; (**d**) perturbation response scanning (PRS) by Gerek et al. [50]; (**e**) statistical coupling analysis (SCA) coupled with a complete single-mutation scan by McLaughlin et al. [56]; (**f**) equilibrium MD by Morra et al. [31]; (**g**) rigid-body MD by Kalescky et al. [47]; and (**h**) equilibrium MD by Kumawat et al. [21]. The neighboring table displays the sequence fragment of the αA helix in the PSD-95 PDZ3 domain with allosteric residues colored accordingly. Note that the colored residues in the table directly correspond to the colored residues in the structural representations on the left.

Next, we will review the studies of three groups that used MD simulations to identify ligand-induced αA allostery in PSD-95 PDZ3. As described above, Morra et al. performed equilibrium MD simulations on both the PTP-BL PDZ2 domain and the PSD-95 PDZ3 domain [31]. While the PSD-95 PDZ3 domain showed less pronounced dynamic changes when compared to PTP-BL PDZ2, the fluctuations in the bound state of PSD-95 PDZ3 notably increased at residues L342 and A343 of the αA helix. In nonequilibrium MD, Kalescky et al. performed rigid-body MD simulations to consider the contribution of each residue to the global protein dynamics [47]. They separated key residues into two groups: switch residues and wire residues. Switch residues were defined as key players in defining a bound or unbound state to “switch on” allosterism in the PDZ3 domain. Sequentially, wire residues propagate the signal. Interestingly, A347 of the αA helix is identified as one of three switch residues. Lastly, Kumawat et al. performed equilibrium MD simulations on the bound and unbound states of PSD-95 PDZ3 [21]. While previous efforts focus on coupled motions or changes in dynamics to define allostery, Kumawat et al. reveal an underlying energetic landscape that couples the binding pocket to distal regions of PSD-95 PDZ3. After noting the general lack of structural and dynamical changes, they describe an energetic landscape that links ligand binding to energetic shifts at D348 and E352 of the αA helix. In summary, various computational techniques have identified αA allostery in PSD-95 PDZ3. Each study described above is summarized in Figure 3. The summarized studies point to the importance of A347 in ligand-induced dynamic allosterism at the αA helix.

#### 1.1.3. Two-Way Communication in Par-6 PDZ

While most efforts have focused on ligand binding as the triggering event in PDZ allosterism, the Par6 PDZ domain has been a unique case. Allostery at the αA helix of the Par6 PDZ domain has been studied in two directions: (1) ligand binding provoking dynamics allosterism at the αA helix and (2) protein–protein interactions at the αA helix altering PDZ binding affinity. In a more traditional understanding of allosterism in the PDZ family, Ho et al. applied rotamerically induced perturbations (RIP) to show that perturbations at the binding site increase fluctuations at the αA helix [59]. More interestingly, several efforts have explored allosteric communication in Par6 PDZ in the opposite direction as well.

Par6 contains a CRIB domain directly adjacent to its PDZ domain. Cdc42 is a protein that regulates the Par6 PDZ domain by binding to the CRIB domain and increasing PDZ binding affinity by ~13 fold [34]. Peterson et al. used NMR spectroscopy to show that when Cdc42 binds to the CRIB domain, it enables a new conformation in which Cdc42 directly interacts with the αA helix of PDZ [34,60]. Notably, interactions between Cdc42 and the αA helix shift the secondary structures that compose the PDZ binding pocket to increase binding affinity. Penkert et al. further support the hypothesis that the shift of secondary structure is key in allowing ligand binding [61]. They considered a unique Par6 PDZ ligand called Pals1. Rather than binding via its C-terminal, Pals1 binds to Par6 PDZ via an internal peptide sequence. Unlike C-terminal ligands, the binding of Pasl1 can induce a conformational shift in the PDZ domain that permits ligand binding. Here, we observe the important role of conformational change in ligand binding to Par6 PDZ. Together, Peterson et al. [34] and Penkert et al. [61] show that the binding of Cdc42 to the surface of the αA helix or the binding of the non-canonical Pals1 ligand can induce this conformational shift. While previous examples point to ligand-induced dynamic allostery at the αA helix, these results suggest that a perturbation at the αA helix can affect ligand binding affinity, ultimately revealing a two-way communication pathway within the Par6 PDZ domain.

In summary, the αA helix is the most recognized region of the PDZ domain that exhibits dynamic allostery. A previous study has primarily explored three PDZ domains: PTP-BL PDZ2, PSD-95 PDZ3, and Par6 PDZ. While the PTP-BL PDZ2 domain has had αA allostery identified by both experimental and computational efforts, the PSD-95 PDZ3 domain lacks consistent experimental results to support αA allostery noted by computational efforts. This may be partially due to the key focus on the αC helix in PSD-95 PDZ3. Studies focusing on the Par6 PDZ domain have been unique to other PDZ domains. Rather than exploring how ligand binding induces dynamic changes, most studies have focused on how protein–protein interactions with distal regions, such as the αA helix, affect binding affinity. Most interestingly, this points towards a symmetrical communication between the αA helix and the binding pocket in the PDZ family. While ligand binding affects the dynamics of the distal αA helix, interactions at the αA helix also regulates binding affinity of ligands.

### 1.2. The αB Lower-Loop

Following the αA helix, the N-terminal region preceding the αB helix has been most frequently identified as a region of dynamic allostery in the PDZ domain. Here, we refer to this region as the αB lower-loop. A previous study has recognized dynamic allostery at the αB lower-loop through various techniques, including NMR, Monte-Carlo sampling, and PRS. Taken together, the various approaches have identified dynamic allostery in PTP-BL PDZ2 and PSD-95 PDZ3.

#### 1.2.1. Agreement between Experimental and Computational Techniques: PTP-BL PDZ2

Taken together, experimental and computational techniques have pointed to the dynamic allostery at the αB lower-loop of PTP-BL PDZ2. In 2004, Fuentes et al. used NMR experiments to show that residues V61, V64, L66, A69, T81, and V85 of the αB lower-loop have increased flexibility upon ligand binding [27]. In a continuation of their original work [35], they performed point mutations to key residues in ligand binding to explore dynamic changes. NMR revealed that I20F and I35V mutants induce dynamic changes to V58, V61, V64, A69, A74, L78, and V85. In another experimental study, Gianni et al. performed double mutant cycles to reveal that R86 is coupled to the ligand [57]. It is worth noting that Fuentes et al. performed these experiments with the RA-GEF2 ligand, which is 15 residues in length. While the residues at positions P_0_ and P_−2_ are primarily responsible for interactions with the PDZ domain, a previous study has revealed that positions P_−4_, P_−5_ and P_−6_ can also directly interact with the PDZ domain at the region of the αB lower-loop [62]. This points to the importance of exploring the dynamics at the αB lower-loop in PDZ complex systems with ligands of longer length, such as RA-GEF2. In addition to experimental techniques, various computational approaches have also identified dynamic allosterism at the αB lower-loop of PTP-BL PDZ2.

As described above, Dhulesia et al. [29], Kong et al. [46], and Morra et al. [31] each performed equilibrium MD simulations to identify dynamic allosterism in PTP-BL PDZ2. With NMR restraints [27,51,52], Dhulesia et al. observed the motion of the αB lower-loop (V61, V64, L66, A69, and T81) increasing and decoupling from the motion of the αB stand upon binding to RA-GEF2 [29]. While Kong et al. and Morra et al. did not restrain their simulations, they also identified significant modulations to PTP-BL PDZ2 upon ligand binding. Kong et al. noted a cluster of residues associated with ligand binding, including G68, A69, L78, T81, and G82 of the αB lower-loop [46]. Morra et al. observed both significant changes in energetic modulations (V61, N62, G63, V64, T70, H71, L72, Q73, A74, V75, E76, T77, L78, and V85) and dynamic fluctuations (V58, L59, A60, V75, E76, T77, and L78) at this region [31]. Cilia et al. performed Monte-Carlo sampling to echo these results [48]. Their sampling identified V58, L59, V61, L66, A74, V75, T77, L78, T81, and V85 as a cluster of dynamically affected residues.

**Figure 4:**
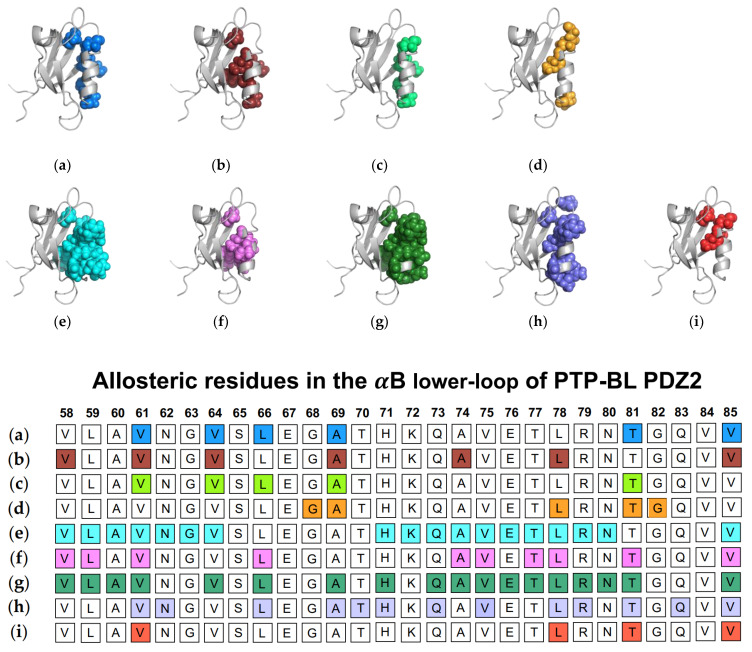
Ligand-induced dynamic allostery at the αB lower-loop in the PTP-BL PDZ2 (PDB ID: 3PDZ) domain as observed in experimental and computational studies. (**a**) NMR chemical shifts by Fuentes et al. [27]; (**b**) point mutations and NMR chemical shifts by Fuentes et al. [35]; (**c**) equilibrium MD with NMR restraints by Dhulesia et al. [29]; (**d**) equilibrium MD by Kong et al. [46]; (**e**) equilibrium MD by Morra et al. [31]; (**f**) Monte-Carlo sampling by Cilia et al. [48]; (**g**) perturbation response scanning (PRS) by Gerek et al. [50]; (**h**) protein structure network (PSN) and elastic network model (ENM) by Raimondi et al. [53]; and (**i**) rigid-residue MD by Kalescky et al. [54]. The neighboring table displays the sequence fragment of the αB lower-loop in the PTP-BL PDZ2 domain with allosteric residues colored accordingly. Note that the colored residues in the table directly correspond to the colored residues in the structural representations shown above.

In addition to traditional simulations, a variety of other computational approaches have been used to explore dynamic allosterism at αB lower-loop in PTP-BL PDZ2. Gerek et al. applied PRS to show that applied forces to specific residues in PTP-BL PDZ2 resulted in a relative displacement of the αB lower-loop (V58, L59, A60, V61, L64, L66, A69, H71, A72, Q73, A74, V75, E76 T77, L78, R79, N80, T81, and V85) [50]. They noted a pathway through S17 of the carboxylate binding loop by which the αB lower-loop was coupled to ligand binding. Combining PSN and ENM, Raimondi et al. identified communication pathways within PTP-BL PDZ2 [53]. They identified a cluster of residues (V61, N62, L66, A69, T70, H71, Q73, V75, L78, R79, T81, Q83, and V85) that are correlated to the RA-GEF2 ligand. Lastly, Kalescky et al. explored the role of each residue in the global dynamics of the protein using rigid-residue molecular dynamics and entropy analysis [54]. They identified several residues including V61, L78, T81, and V85 that have a key role in the allosteric network. Each study described above is summarized in Figure 4. The summarized works point to the importance of V61, V64, L66, A69, A74, L78, T81, and V85 in ligand-induced dynamic allosterism at the αB lower-loop.

#### 1.2.2. Computational Conclusions Lacking Experimental Support: PSD-95 PDZ3

While computational results pointing to ligand-induced dynamic allosterism in the αB lower-loop of PTP-BL PDZ2 have been validated by NMR experiments, computational results pointing to ligand-induced dynamic allosterism in the αB lower-loop of PSD-95 PDZ3 have not been so consistent. As described above, original experimental work on the PSD-95 PDZ3 domain specifically noted the lack of conformational changes to the protein backbone upon ligand binding [17]. Since then, various computational studies have suggested that the αB lower loop may have a role in the propagation of dynamic allostery.

**Figure 5:**
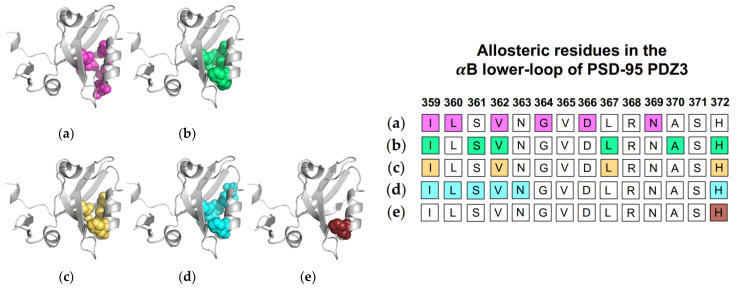
Ligand-induced dynamic allostery at the αB lower-loop in the PSD-95 PDZ3 (PDB ID: 1BE9) domain as observed in experimental and computational studies. (**a**) Conservative-mutation correlation analysis (CMCA) by Du et al. [58]; (**b**) perturbation response scanning (PRS) by Gerek et al. [50]; (**c**) statistical coupling analysis (SCA) coupled with a complete single-mutation scan by McLaughlin et al. [56]; (**d**) equilibrium MD by Morra et al. [31]; and (**e**) rigid body MD by Kalescky et al [47]. The neighboring table displays the sequence fragment of the αB lower-loop in the PSD-95 PDZ3 domain with allosteric residues colored accordingly. Note that the colored residues in the table directly correspond to the colored residues in the structural representations on the left.

Normal mode analysis (NMA) of the PSD-95 PDZ3 domain showed a shift of the αB helix that more widely opened the binding pocket to permit ligand binding [22]. Additionally, CMCA identified a correlated network including various residue at the αB lower-loop (I359, L360, V362, G364, D366, and N369) [58]. PRS has revealed that applied forces on PSD-95 PDZ3 resulted in relative displacements of the αB lower loop and pointed to I359, S361, V362, L367, and H372 as key residues in the pathway of allosteric propagation [50]. Taken together, complete single-mutation scan coupled with SCA identified residues I359, V362, L367, and H372 as being significantly correlated with ligand binding [56]. As previously described, Morra et al. performed equilibrium MD simulations to reveal significant energetic modulations to PSD-95 PDZ3 at residues I359, L360, S361, V362, N363, and H72 upon ligand binding [31]. Finally, Kalescky et al. performed non-equilibrium rigid-body MD simulations to consider the contribution of each residue to global protein dynamics [47]. H372 was identified as one of five “wire” residues that are responsible for the propagation of allosteric signal. It should be noted that while the approaches described the above point to dynamic allosterism at the αB lower loop, other computational studies on PSD-95 PDZ3 failed to recognize this allosteric region [21,49,57]. Each study described above is summarized in Figure 5. The summarized studies point to the importance of I359, V362, L367, and H372 in ligand-induced dynamic allosterism at the αB lower loop.

### 1.3. The αC Helix in PSD-95 PDZ3

While the PDZ family has a highly conserved fold, the PSD-95 PDZ3 domain has a unique αC helix at the C-terminal of the domain. Due to the fact that this added helix is not a part of the traditional PDZ fold, it drew attention for researchers to explore its functional role. Taken together, experimental and computational techniques have identified this unique helix as a key player in allosteric regulation of ligand binding. In 2009, Petit et al. made the first effort to explore the role of the αC helix by comparing the wild-type PSD-95 PDZ3 to an αC-truncated PSD-95 PDZ3 using NMR [36]. While the removal of the αC helix (residues 396-402) had little effect on the fold of PDZ3, it reduced the binding affinity by 21-fold. Furthermore, the αC-truncated PSD-95 PDZ3 showed a global reduction in S2axis, signifying a global increase in side-chain flexibility and, thus, side-chain entropy. While side-chain flexibility was increased by truncation of the αC helix, ligand binding to the αC-truncated system reduced and restored typical side-chain flexibility. This entropic penalty upon ligand binding can explain the 21-fold reduction in binding affinity of the truncated system; ultimately, Petit et al. concluded that ligand binding is entropically driven in PSD-95 PDZ3. In efforts to explore how nature regulates this process, Zhang et al. applied NMR experiments to study that a phosphorylated PSD-95 PDZ3.59 PSD-95 PDZ3 has more than 10 phosphorylation sites, including Y397 on the αC helix [37]. Comparing the Y397-phosphorylated PSD-95 PDZ3 to the wild-type PSD-95 PDZ3, they observed that Y397-phosphorylated PSD-95 PDZ3 has (1) a rapid equilibrium of folded and unfolded αC helix; (2) increased entropy; and (3) reduced binding affinity. Their study reinforced the idea of the αC helix having a key role in ligand binding while also revealing the mechanism by which nature regulates the entropy of the helix and, in turn, the global entropy of PSD-95 PDZ3. Most recently, Bozovic et al. incorporated an azobenzene-based photoswitch to the αC helix of PSD-95 PDZ3 to control the conformation of the αC helix [63]. Interestingly, they calculated a positive and negative binding enthalpy for the folded αC and unfolded αC, respectively. Their results echo Petit et al.’s [36] by reinforcing the idea of entropically driven ligand binding.

**Figure 6:**
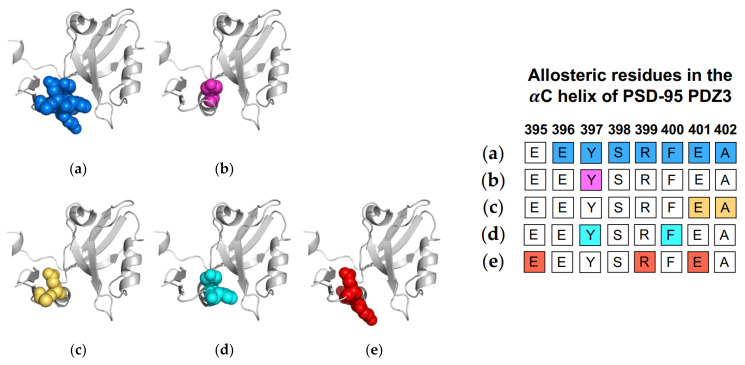
Ligand-induced dynamic allostery at the αC helix in the PSD-95 PDZ3 domain (PDB ID: 1BE9) as observed in experimental and computational studies. (**a**) NMR chemical shifts by Petit et al. [36]; (**b**) NMR chemical shifts by Zhang et al. [37]; (**c**) equilibrium MD by Morra et al. [31]; (**d**) rigid-body MD by Kalescky et al. [47]; and (**e**) equilibrium MD by Kumawat et al [21]. The neighboring table displays the sequence fragment of the αC helix in the PSD-95 PDZ3 domain with allosteric residues colored accordingly. Note that the colored residues in the table directly correspond to the colored residues in the structural representations on the left.

Computational efforts also have supported the allosteric role of the αC helix in ligand binding. Morra et al. performed equilibrium MD simulations to identify a significant correlation between fluctuations and core energy at the αC helix (E401 and A402) [31]. Rigid-body MD simulations performed by Kalescky et al. identified Y397 and F400 on the αC helix as a switch residue (“switch on” allosterism) and a wire residue (propagate allosterism), respectively [47]. Three years later, Kumawat et al. used classical MD simulations to compare residual energies in the bound and unbound states of PSD-95 PDZ3 [21]. They show that ligand binding propagates an energetic change at the αC helix (E395, R399, and E401) via electrostatic interaction population through a shift in the population of hydrogen bonds within PSD-95 PDZ3. Like the Par6 PDZ domain, the αC helix in PSD-95 PDZ3 has been shown to have a symmetrical pathway to the binding pocket in dynamic allosterism. Petit et al. [36], Zhang et al. [37], and Bozovic et al. [63] all point to the role of the αC helix in regulating binding affinity. Oppositely, other groups [21,31,47,59] have shown that ligand-binding propagates an allosteric signal to the αC helix. Each study described above is summarized in Figure 6. The summarized works point to the importance of Y397 in dynamic allosterism at the αC helix in PSD-95 PDZ3.

## 2. Perspective

While 268 PDZ domains have been identified in the human proteome [7], efforts exploring dynamic allosterism in the PDZ family have focused on PTP-BL PDZ2, PSD-95 PDZ3, and Par6 PDZ. Focusing on these well-studied PDZ domains, various groups have revealed three regions of the PDZ domain that play a key role in dynamic allosterism. These regions are referred to here as the αA helix, the αB lower-loop, and the αC helix. The purpose of this review is to explore dynamic allosterism at these three key regions in the three most well-studied PDZ domains. We show that previous experimental studies have pointed to the role of dynamic allostery at the αA helix of Par6 PDZ but have not identified the αB lower-loop. Furthermore, our review shows that ligand-induced dynamic allostery in the PTP-BL PDZ2 domain has been explored using both experimental and computational techniques to identify allosterism at the αA helix and the αB lower-loop. Oppositely, efforts identifying dynamic allostery at the αA helix and the αB lower-loop on the PSD-95 PDZ3 domain have primarily used computational techniques and have not been consistently verified by experimental approaches. Instead, experimental techniques have focused on the αC helix of PSD-95 PDZ3. Taken together, these efforts have pointed to dynamic allostery at the αA helix and the αB lower loop in the PTP-BL PDZ2 domain and the PSD-95 PDZ3 domain. Interestingly, our review reveals some level of agreement between the key allosteric residues in PTP-BL PDZ2 and PSD-95 PDZ3.

**Figure 7:**
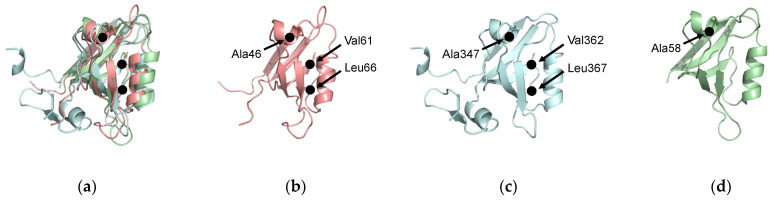
Evolutionarily conserved residues. (**a**) Structural alignment of PTP-BL PDZ2 (pink), PSD-95 PDZ3 (blue), and PICK1 PDZ (green). A46, A347, and A58 have been identified as key residues in dynamic allosterism at the αA helix. V61, L66, V362, and L367 have been identified as key residues in dynamic allosterism at the αB lower loop. (**b**) PTP-BL PDZ2. (**c**) PSD-95 PDZ3. (**d**) PICK1 PDZ.

From the above, we have listed residues consistently identified as key players in dynamic allosterism in each PDZ domain. For PTP-BL PDZ2, these residues include V40, A45, A46, V61, L66, A69, L78, and T81. For PSD-95 PDZ3, these residues include A347, V362, L367, H372, and Y397. Interestingly, some of the key residues involved in dynamic allosterism have structural agreement between the two domains. The structural alignment between PTP-BL PDZ2 (pink) and PSD-95 (blue) is shown in Figure 7a. Despite the low sequence identity between (36.99%) between PTP-BL PDZ2 and PSD-95 PDZ3, the fold is highly conserved between the two domains so that a structural alignment between the two domains can show an agreement between key residues in each domain. For example, various efforts have specifically pointed to the importance of A46 and A347 in PTP-BL PDZ2 and PSD-95 PDZ3, respectively. We show that these residues are evolutionarily conserved as they are structurally aligned on the αA helix. Furthermore, our recent study also points to the importance of A58 of the PICK1 PDZ domain in the dynamic allosterism at the αA helix [64]. Interestingly, A58 of PICK1 PDZ (green) also structurally aligns with A46 and A347. Notably, A46, A347, and A58 are in direct proximity to the carboxylate-binding loop that forms interactions with the ligand. This conserved alanine residue may be a key player in the transduction of signals between the αA helix and the binding pocket. Like the highly conserved alanine residue on the αA helix, many of the key residues involved in dynamic allosterism on the αB lower-loop also have structural agreement between PTP-BL PDZ2 and PSD-95 PDZ3. Various efforts have specifically pointed to the importance of V61 and L66 in PTP-BL PDZ2 and V362 and L367 in PSD-93 PDZ3. As shown in Figure 7, V61 and L66 of PTP-BL PDZ2 (pink) and V362 and L367 of PSD-95 (blue) are structurally aligned, respectively. Not only are these residues conserved, but they also have both been identified as key players in dynamic allosterism on the αB lower-loop.

In Lockless and Ranganathan’s original study [20], they propose that multiple sequence alignment can be used to identify residues involved in allosteric pathways because allosterism is evolutionarily conserved. Here, we work backwards to support this hypothesis. After considering more than a dozen studies exploring dynamic allosterism in PTP-BL PDZ2 and PSD-95 PDZ domains, we have noted three residues that have been consistently identified in the allosteric network: A46/A347, V61/V362, and L66/L367 on PTP-BL PDZ2 and PSD-95 PDZ3, respectively. Interestingly, these residues occur at evolutionarily conserved positions (alanine, valine, and leucine). In summary, a thorough review of the previous efforts to identify allosterism in the PDZ family point back to Lockless and Ranganathan’s original hypothesis that allosteric networks are evolutionarily conserved.

While some consistently identified allosteric residues within PTP-BL PDZ2 and PSD-95 PDZ3 are in good agreement between the two domains, this is not the case for all key residues. For example, experimental and computational efforts consistently point to V40 and A45 of the αA helix as key allosteric residues in the PTP-BL PDZ2 domain while the structurally equivalent I341 and P346 of PSD-95 PDZ3 are not. Similarly, A69, L78, and T81 of the αB lower-loop have been identified as key allosteric residues in the PTP-BL PDZ2 domain but their structural equivalents in PSD-95 PDZ3 are not. H372 of the αB lower-loop in PSD-95 PDZ3 is a key allosteric residue. While two computational studies identify the structurally equivalent His71 of PTP-BL PDZ2 as a key residue, it has not been consistently identified over all previous studies. These results suggest that while pieces of the allosteric network are conserved in the PDZ family, the dynamic allostery within each PDZ domain remains unique. Our previous study explored ligand-induced dynamic allosterism within the PICK1 PDZ domain [64]. This study was unique because it considered the effects of two different natural ligands: AMPA receptor GluR2 and the Dopamine Transporter (DAT). We show that the different ligands induce different dynamic allostery within the same PDZ domain. We suspect that PDZ domains with multiple binding partners may have coexisting allosteric networks corresponding to each binding partner. Other previous studies have not yet explored this possibility. Most groups exploring PTP-BL PDZ2 have used RA-GEF as the ligand of interest [27,31,46,48,53,59]. Only a few groups have considered APC [57]. Similarly, CRIPT has been primarily used as the ligand of interest to study PSD-95 PDZ3 [21,31,47,57]. To the best of our knowledge, no attention has yet been placed into exploring the possibility of how different ligands binding to either PTP-BL PDZ2 or PSD-95 PDZ3 may affect dynamic allosterism in the PDZ domain.

Having identified dynamic allosterism in the PDZ family, we are left with an apparent question: How does the PDZ domain utilize dynamic allosterism in its biological function? Various efforts have begun to address this mystery. Specifically, in the Par6 PDZ domain, interactions at the αA helix have shown to alter the binding affinity of the PDZ domain through allosteric communications [63]. Additionally, the PTP-BL PDZ1 domain can form interactions with the surface of the αA helix of the PTP-BL PDZ2 domain to alter the binding specificity of PDZ2 [45]. Interestingly, after observing the propagation of allosterism to the αC helix of the PSD-95 PDZ3 domain, Kalescky et al. noted that “the allosteric effects propagate to the termini regions, possibly leading to a global response in terms of spatial arrangement of these domains on binding with the effector ligands/proteins” [47]. Ultimately, these results and hypotheses point to the role of dynamic allosterism in the PDZ family as a regulator in higher-order systems of protein–protein interactions. While this may be true, most studies considering allosterism in the PDZ family have studied the PDZ domain isolated from its neighboring domains and linkers. Exploring only the isolated PDZ domain may be hindering our understanding of the role of dynamic allosterism as a regulator of biological function. In 2011, Zhang et al. went further to study a system of the PSD-95 that included the PDZ3 domain and the SH3 domain connected by a linker [37]. They noted that a perturbation at Tyr397 not only affects the PDZ3 domain [36,37,63] but also alters the dynamic interactions between PDZ3 and SH3. These results point to the importance of not limiting our understanding of allosterism within a single PDZ domain but instead exploring the role of dynamic allosterism in multidomain constructs of the PDZ family.

## Data Availability

Not applicable.
